# Legume Consumption and All-Cause and Cardiovascular Disease Mortality

**DOI:** 10.1155/2017/8450618

**Published:** 2017-11-02

**Authors:** Hua Li, Jinmeng Li, Yegen Shen, Jie Wang, Depu Zhou

**Affiliations:** ^1^Department of Cardiology, The Second Affiliated Hospital and Yuying Children's Hospital of Wenzhou Medical University, Xueyuanxi Road, No. 109, Wenzhou, Zhejiang 325000, China; ^2^Department of Pharmacy, School of Pharmaceutical Science, Wenzhou Medical University, Wenzhou, China

## Abstract

**Background:**

Legume consumption is suggested to have protective effects against cardiovascular disease (CVD) mortality in the general population, but the results have been equivocal. We conducted a meta-analysis of prospective cohort studies to assess the association between legume consumption and risk of CVD mortality and all-cause mortality.

**Methods and Results:**

Medline (via Ovid) and EMBASE (via Ovid) databases were searched through April 2017 to identify eligible studies. The two authors independently extracted the data and the adjusted relative risks (RRs) and 95% confidence intervals (CIs) were pooled by using a random-effects model. A total of 6 studies were identified, including the sizes of participants ranging from 23,601 to 59,485 with a sum of 21,8997. Comparing the highest category with the lowest, the pooled RR (95% CI) was 0.96 (0.86–1.06) for CVD mortality and 0.93 (0.87–0.99) for all-cause mortality.

**Conclusions:**

Results from the current study show that high legume intakes are associated with lower risk of all-cause mortality. In consideration of the small number of studies, the evidence for assessing relationship between legumes intake and risk of all-cause mortality remains inclusive and warrants further study in the future. Further, consuming legumes does not increase the risk of CVD mortality.

## 1. Introduction

Cardiovascular disease (CVD) is the leading cause of death worldwide, accounting for nearly one-third of all deaths globally [1, 2]. This constitutes 17% of overall national health expenditures in the US [2, 3]. To reduce the burden, the prevention of CVD morbidity and mortality has been increasingly prioritized in public health [4]. It is well accepted that healthy diet has beneficial effects on decreasing the burden of CVD incidence and mortality [5].

Legumes include peas, beans, lentils, and peanuts and are a rich source of phytosterols and dietary fiber [6, 7]. Previous dietary trials report that the consumption of vegetable protein instead of animal protein lowers blood cholesterol levels, which may lead to lowering the risk of CVD [8]. Many epidemiologic studies [9–14] have assessed the relationship between legume intake and all-cause mortality and CVD mortality and most found a positive association. But the magnitudes of the association varied between studies.

With accumulating evidence, we conducted a meta-analysis to assess the association between legume intake and risk of CVD mortality and all-cause mortality.

## 2. Methods

### 2.1. Literature Search

We performed a systematic search in Medline (via Ovid) and EMBASE (via Ovid) databases in April 2017 to identify studies regarding the association between legume intake and risk of CVD mortality and all-cause mortality. And we updated the literature search of Medline (via Ovid) and EMBASE (via Ovid) database on June 2017 to make sure our study results were up-to-date. We used the following search terms: “Legume”, “vegetable proteins”, “soy products”, “cardiovascular disease mortality” AND “mortality”. References from the relevant review were examined to identify relevant articles.

### 2.2. Study Selection

Literatures were included in the meta-analysis if (1) the study design was a cohort study (i.e., not review articles, comments, or conference abstracts); (2) the exposure was legume intake; (3) the end points were CVD mortality and all-cause mortality; and (4) risk estimate and the corresponding 95% confidence intervals (CI) were reported. A study must meet all the four inclusion criteria for inclusion. Articles were identified by screening of titles or (and) abstracts and full-text articles of the screened studies were later assessed by two authors (X.Q.G and H.L). The differences in view regarding the selection were resolved by discussion. If one study had multiple publications, we selected the largest sample of articles [15].

The agreement between the two authors (X.Q.G and H.L) was 99.4% for the first screening and 100% for the full-text articles.

### 2.3. Data Extraction

We extracted the following information using a standardized extraction form: (1) the author name; (2) publication year; (3) regions and cohort details; (4) number, sex, and age of participants and number of cases; (5) mean length of follow-up; (6) outcome; and (7) confounder adjustment and RR from multivariable adjustment model for the highest versus the lowest category of legume intake with corresponding 95% CI. To get missing data and clarify unclear data, we contacted the authors and coauthors by email.

### 2.4. Statistical Analysis

We used the RR for the highest level of legume consumption compared with the lowest to assess the association between legume intake and risk of CVD mortality and all-cause mortality [16]. In this meta-analysis, the hazard ratio was considered as a surrogate measure of RR, and the OR was transformed into RR by the formula RR = OR/[(1 − Po)+(Po × OR)], where Po is the incidence of the end point in the control group [17]. Forest plots were made to visually assess the RR and corresponding 95% CI across studies [18, 19]. We calculated *Q* test (significance level of *P* < 0.10) and the *I*^2^ statistic (ranging from 0% to 100%) to examine the heterogeneity across studies. The DerSimonian and Laird inverse-variance-weighted random-effects model was used to pool the RR [20].

Subgroup analyses were performed to evaluate the potential effect modification of these variables (i.e., female versus male) on outcomes. We also conducted sensitivity analysis by omitting one article at every turn to investigate whether overall risk estimates would be heavily affected by a single study [21].

Review Manager software (version 5.3; the Nordic Cochrane Centre, Copenhagen, Denmark) was used for the statistical analyses, which is provided by Cochrane collaboration. A two-sided *P* value of less than 0.05 was considered statistically significant [15].

## 3. Results

### 3.1. Literature Identification and Search

**Figure 1:**
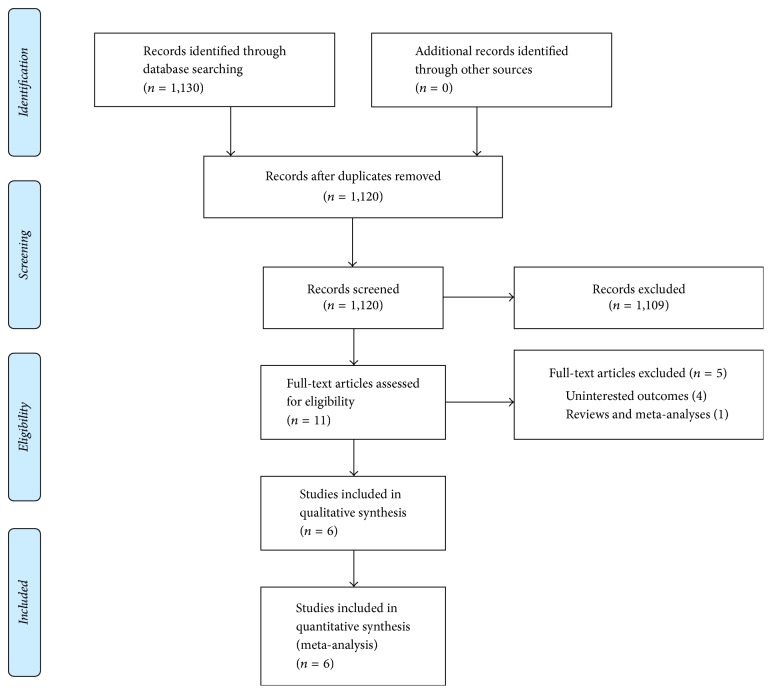
Flowchart of literature search and study selection.

Detailed flowchart of articles for inclusion is presented in Figure 1. We identified 1130 potential relevant articles from the Medline (via Ovid) and EMBASE (via Ovid) databases. After removing duplicated articles and review of the titles or (and) abstracts, the 11 articles were selected. The remaining articles were further checked for eligibility. Finally, a total of 6 studies [9–14] were eligible for this meta-analysis and the full list of publications is shown in Table 1.

### 3.2. Study Characteristics

The characteristics of each cohort studies are shown in Table 1. The studies were published between 2005 and 2016. With regard to the study region, most studies were conducted in Asia countries (*n* = 5), and the others were in European (*n* = 1) and North America (*n* = 1). Five studies included participants with both genders and one study included only males. The length of follow-up ranged from 10 to 15 years, with an average of 12.0 years. The sizes of cases ranged from 175 to 3,291, with an average of 1,246. The sizes of participants ranged from 23,601 to 59,485 with a sum of 21,8997.

### 3.3. Legume Consumption with Cardiovascular Disease Mortality

**Figure 2:**
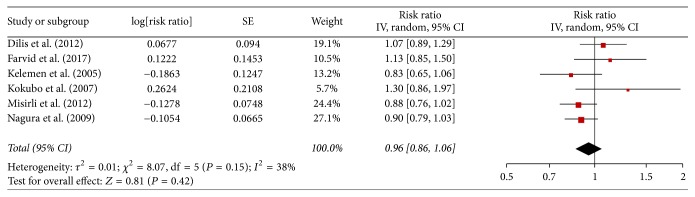
Meta-analysis of observational studies on legume consumption and risk of CVD mortality.

Across 6 studies with data on cardiovascular disease mortality, the RR from individual studies ranged from 0.83 to 1.30. All of them do not report statistically significant association between legume consumption and the risk of cardiovascular disease mortality. The pooled adjusted RR was 0.96 (0.86–1.06; Figure 2), and moderate heterogeneity was found among studies (*I*^2^ = 38%; *P*_heterogeneity  _ = 0.42).

The sensitivity analysis was performed by omitting one study in each time to show statistically significant or marginally significant results; the pooled RR varied from 0.93 (95% CI, 0.83–1.04) to 0.99 (95% CI, 0.86–1.13). We conducted stratified analyses by geographic area, the number of participates, and sex (Table 2). Egger test and funnel plot were not conducted for the small number of studies.

### 3.4. Legume Consumption with All-Cause Mortality

**Figure 3:**
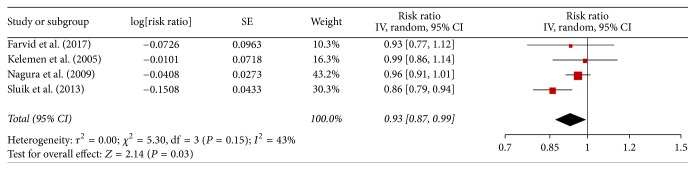
Meta-analysis of observational studies on legume consumption and risk of all-cause mortality.

There 4 studies reported association between legume consumption and the risk of all-cause mortality. The pooled adjusted RR was 0.93 (0.87–0.99; Figure 3), and moderate heterogeneity was found among studies (*I*^2^ = 43%; *P*_heterogeneity_ = 0.03).

The sensitivity analysis also was conducted and the pooled RR ranged from 0.91 (95% CI, 0.83–1.00) to 0.96 (95% CI, 0.92–1.01). Because of the small number of studies, subgroup analysis and funnel plot were not performed.

## 4. Discussion

Epidemiologic studies on the health effects of legume consumption have accelerated recently, our meta-analysis of six-cohort studies including 21,8997 participates show inverse associations between legume consumption and all-cause mortality. We do not find a significant relationship between legume consumption and cardiovascular disease mortality. And moderate heterogeneity was found across studies.

This is the first meta-analysis specifically evaluated the association between legume consumption and cardiovascular disease mortality and all-cause mortality. There are a lot of strengths in this meta-analysis. A major advantage of this meta-analysis is that all included studies were of a prospective cohort design, which can reduce the likelihood of selection bias and recall bias [22, 23]. It is difficult to perform a long-term, randomized controlled trial on legume consumption and CVD mortality. Moreover, the studies included in our study had a large sample participants and long-term follow-up time, which can potentiate the statistical power to evaluate the relationship [24]. For instance, the length of follow-up ranged from 10 to 15 years (average of 12.0 years) and the sizes of participants ranged from 23,601 to 59,485 with a sum of 21,8997. In addition, sensitivity analyses showed that our result was not substantially affected by single studies, which shows our findings were robust.

Some limitations warrant discussion. First of all, our results were based on observational studies, and the observed association may be affected by possibility of residual confounding [25]. Second because of the small number of studies, subgroup analysis, funnel plot, and dose-response analysis were not performed [26]. Third, FFQ used in the most primary studies had high reproducibility but low-to-moderate validity for the estimation of the legume intakes [27, 28]. Consequently, misclassification may be to dent the association [29].

It has been thought that legume consumption may reduce the risk of CVD mortality.

Legumes are rich in phytosterols, which can reduce serum total cholesterol and low-density lipoprotein cholesterol and a significant increase in high density lipoprotein cholesterol [6, 7, 30]. But whether dietary cholesterol consumption significantly influences the lipid profile and subsequently is associated with the risk of CVD and CVD mortality is still unclear [31].

For all-cause mortality, the result shows inverse associations between legume consumption and all-cause mortality. But the sensitivity analysis was conducted and the result is not stable. In consideration of the small number of studies, the evidence for assessing relationship between legumes intake and risk of all-cause mortality remains inclusive and warrants further study in the future.

## 5. Conclusion

In conclusion, results from the current study show that high legume consumption is associated with lower risk of all-cause mortality. In consideration of the small number of studies, the evidence for assessing relationship between legumes intake and risk of all-cause mortality remains inclusive and warrants further study in the future. And our results do not support an increase in the risk of CVD mortality in the general population with legume consumption.

## Figures and Tables

**Table 1:** Characteristics of observational studies of legume consumption and risk of mortality included in this meta-analysis.

Study: first author, year	Country cohort details	Age (years)	Sex	Number of cases/number of control (participants)	Mean follow-up (years)	Exposure assessment	Outcome	Adjustment for covariates
Kelemen et al. (2005)	IWHS (USA)	55–69	M	739/29,017	15	FFQ	CHD mortality/total mortality	Age, total energy, polyunsaturated fat, monounsaturated fat, trans-fat, total fiber, dietary cholesterol, dietary methionine, alcohol, smoking, activity level, BMI, history of hypertension, saturated fat, postmenopausal hormone use, multivitamin use, vitamin E supplement use, education, family history cancer
Kokubo et al. (2007)	JPHC (Japan)	40–59	M and F	175/40,462	12.5	FFQ	CVD mortality	Age, sex, smoking, alcohol use, BMI, history hypertension or DM, medication use for hypercholesterolaemia, education level, sports, dietary intake of fruits, vegetables, fish, salt and energy, menopausal status for women
Dilis et al. (2012)	EPIC (European)	20–86	M and F	636/23,929	10	FFQ	CHD mortality	Age, BMI, height, physical activity, years of schooling and energy intake entered as continuous variables. Alcohol consumption, smoking status and arterial blood pressure, potatoes, vegetables, fruits and nuts, dairy foods, cereals, meat, fish, eggs, sweets, nonalcoholic beverages, saturated lipids, monounsaturated lipids, polyunsaturated lipids, monounsaturated: saturated lipid ratio
Farvid et al. (2017)	The Golestan Cohort Study (Iran)	36–85	M and F	3,291/42,403	11	FFQ	CVD mortality/total mortality	Age, sex, ethnicity, marital status, residency, smoking, opium use, alcohol, BMI, systolic blood pressure, occupational physical activity, family history of cancer, wealth score, medication, energy intake
Misirli et al. (2012)	EPIC (European)	25–67	M and F	395/23,601	10.6	FFQ	CVD mortality	Age, education, smoking status, BMI, physical activity, hypertension, DM, energy intake
Nagura et al. (2009)	JACC (Japan)	40–79	M and F	2,243/59,485	12.7	FFQ	CVD mortality/total mortality	Age, sex, BMI, smoking status, alcohol intake, hours of walking, hours of sleep, education, perceived mental stress, cholesterol intake, SFA intake, n-3 fatty acids intake, Na intake, history of hypertension, history of diabetes, fruit and vegetable intakes

IWHS, Iowa Women's Health Study; DM: diabetes mellitus; JPHC, Japan Public Health Center-based; EPIC, European Prospective Investigation into Cancer and Nutrition; JACC, Japan Collaborative Cohort Study; M: male; F: female; BMI: Body Mass Index; FFQ: food frequency questionnaire; CVD: cardiovascular disease.

**Table 2:** Stratified analyses of mortality associated with legume consumption.

Group	Number of studies	RR (95% CI)	*P* (heterogeneity)	*I* ^2^ (%)
Total	6	0.96 [0.86, 1.06]	0.42	38
Geographic area				
North America	1	0.83 [0.65, 1.06]	/	/
Asia	3	1.03 [0.83, 1.29]	0.76	53
Europe	2	0.96 [0.80, 1.16]	0.69	62
Number of participates				
<30,000	3	0.93 [0.80, 1.07]	0.31	44
≥30,000	4	1.03 [0.83, 1.29]	0.76	53
Sex				
Men	2	0.91 [0.77, 1.07]	0.25	0
Women	1	0.72 [0.50, 1.05]	/	/
